# How can physiology best contribute to wildlife conservation in a warming world?

**DOI:** 10.1093/conphys/coad038

**Published:** 2023-06-03

**Authors:** Frank Seebacher, Edward Narayan, Jodie L Rummer, Sean Tomlinson, Steven J Cooke

**Affiliations:** School of Life and Environmental Sciences A08, University of Sydney, NSW 2006, Australia; School of Agriculture and Food Sciences, The University of Queensland, St. Lucia QLD4072, Australia; College of Science and Engineering and ARC Centre of Excellence for Coral Reef Studies, James Cook University, Townsville QLD 4810, Australia; School of Biological Sciences, University of Adelaide, SA 5000, Australia; Fish Ecology and Conservation Physiology Laboratory, Department of Biology, Carleton University, Ottawa, ON K1S 5B6, Canada

**Keywords:** Conservation Standards, climate warming, environmental monitoring, thermal sensitivity, plasticity, species distribution models, food webs

## Abstract

Global warming is now predicted to exceed 1.5°C by 2033 and 2°C by the end of the 21st century. This level of warming and the associated environmental variability are already increasing pressure on natural and human systems. Here we emphasize the role of physiology in the light of the latest assessment of climate warming by the Intergovernmental Panel on Climate Change. We describe how physiology can contribute to contemporary conservation programmes. We focus on thermal responses of animals, but we acknowledge that the impacts of climate change are much broader phylogenetically and environmentally. A physiological contribution would encompass environmental monitoring, coupled with measuring individual sensitivities to temperature change and upscaling these to ecosystem level. The latest version of the widely accepted *Conservation Standards* designed by the Conservation Measures Partnership includes several explicit climate change considerations. We argue that physiology has a unique role to play in addressing these considerations. Moreover, physiology can be incorporated by institutions and organizations that range from international bodies to national governments and to local communities, and in doing so, it brings a mechanistic approach to conservation and the management of biological resources.

## Teaser

Changing environmental conditions first and foremost affect the physiology of animals. Therefore, physiological responses to climate warming should be included in the assessment of conservation needs and strategies. An integrated conservation approach, including physiology, ecology, and genetics, should lead to the most robust conservation outcomes.

## Introduction

It is now likely that global warming will exceed 2°C by the end of the 21st century (Masson-Delmotte *et al.,* 2021; Pörtner *et al.,* 2022). Increases in CO_2_ emissions have slowed (LeQuéré *et al.,* 2019), but mitigation strategies are presently insufficient to limit global average temperature increases to 1.5°C or even 2°C (Masson-Delmotte *et al.,* 2021). Additionally, human activity may have already emitted sufficient carbon into the atmosphere to cause warming well beyond 1.5°C without any further emissions (Matthews and Wynes, 2022).

Anthropogenic climate change is having and will continue to have impacts on wildlife from individuals to ecosystems (Moore and Schindler, 2022). Global mean increases of 2°C or even 1.5°C are associated with much greater variation at regional and local levels, as well as with increasing frequencies of extreme events (Meehl and Tebaldi, 2004;Wedler *et al.,* 2023). Hence, while increases of 2°C may sound benign, this large-scale mean hides much greater variation at smaller scales, which are potentially damaging to wildlife (Kingsolver and Buckley, 2015). Indeed, distributions and phenology of life-history events have already shifted in many species (Chen *et al.,* 2011; Bellard *et al.,* 2012). Increasing mean temperatures are also accompanied by an increasing frequency of extreme events such as heat waves, which can have pronounced effects on animal physiology, resulting either from temperature increases directly or from changes to other environmental factors such as rainfall and the hydric environment (Meehl and Tebaldi, 2004; Conradie *et al.,* 2020; Schoen *et al.,* 2021). For example, there is an increase in the temperature of the hottest days of the year from ~ 2°C to ~ 4°C associated with mean global temperature increases of 1.5°C and 2°C, respectively (Lee *et al.,* 2023). These increases can have detrimental physiological effects particularly for species with a low thermal safety margin (Sinclair *et al.,* 2016; Pollock *et al.,* 2021). Changes in extreme temperatures are paralleled by a predicted increase in species loss under the 2°C warming scenario (Lee *et al.,* 2023). Environmental variability is characteristic of all habitats, and ecosystems typically undergo cycles of disturbance and recovery (Paine *et al.,* 1998). As a result, environmental variability exerts a selection pressure that can drive adaptation or plasticity so that disturbance–recovery cycles have little long-term effects (Paine *et al.,* 1998; Moore and Schindler, 2022). However, ecosystems are resilient only up to a tipping point beyond which dynamics change irreversibly and a new status quo emerges (Gaucherel *et al.,* 2017). Anthropogenic climate change and the consequent global warming are now increasing the likelihood of reaching tipping points as warming increases beyond a global average of 1.5°C (Armstrong-McKay *et al.,* 2022; Solé and Levin, 2022).

How human societies function is tightly coupled to ecological systems (Haines-Young and Potschin, 2010), and ecological changes resulting from climate warming impact the services that ecosystems provide to support human life (Burke *et al.,* 2015). The nexus between human and ecological systems is particularly pronounced in food supply. On the one hand, human food systems rely on suitable environmental conditions to grow or locate food species for agriculture or wild harvest (Ortiz *et al.,* 2008; Nardone *et al.,* 2010; Pecl *et al.,* 2017). Climate change has already affected global food production negatively (Pörtner *et al.,* 2022), and the impacts of changing climates may be more complex than just volumes of production. Global fisheries, for example, are vulnerable not just in the volume of fish caught but also in the nutritional quality of the fish caught, with 40% of fisheries displaying high vulnerability to climate-induced nutritional decline (Maire *et al.,* 2021). On the other hand, agriculture and harvesting of natural populations alter the physical environment and biodiversity (Tilman, 1999). Food systems are now one of the most important contributors to climate change and account for a third of anthropogenic greenhouse gas emissions (Zurek *et al.,* 2022).

This *Perspective* is not the first to make the case that physiology can direct conservation in the context of climate change (Helmuth *et al.,* 2005; Helmuth, 2009; Feder, 2010; Burraco *et al.,* 2020; Lefevre *et al.,* 2021). However, our purpose here is to emphasize the role of physiology in the light of the latest assessment of climate warming by the Intergovernmental Panel on Climate Change (Pörtner *et al.,* 2022) and to position physiology within contemporary conservation programmes, particularly with respect to the *Conservation Standards (CS)*. We focus in particular on thermal responses to climate warming; we acknowledge that climate change is far more complex (Pörtner *et al.,* 2022), but a detailed review is beyond the scope of this article. Nonetheless, the approach we describe here can be applied to different aspects of climate change beyond warming. Physiology has a unique role to play because it is at the interface between environment and organisms. Any change in the environment will first and foremost affect physiology, and the physiological responses will then impact fitness and ecology (Ricklefs and Wikelski, 2002). We outline how physiology can be incorporated into conservation programmes, and we provide examples of how knowledge of thermal physiology can improve conservation strategies. Our examples are from animals, but the principal points we make can be applied to any organism.

## How can physiology inform conservation?

Climate warming causes changes in mean temperatures and in temperature variation, with an increased frequency of extreme events (Vasseur *et al.,* 2014). It is likely that there is a gradient of responses for different species within ecosystems, where those with greater resilience to temperature changes persist better in the face of climate warming, thus altering the species composition within ecosystems (Zoller *et al.,* 2023). These high-level changes are underpinned by thermal responses of individuals, which scale up to populations, species and communities (Sentis *et al.,* 2015). Understanding and predicting the ecological impacts of climate warming therefore requires resolution at different scales: from individuals to communities, and from microhabitats to landscape characteristics. A conservation physiology programme will be invaluable by integrating different biological and geographical scales and by integrating with existing conservation actions (Cooke *et al.,* 2021).

**Figure 1:**
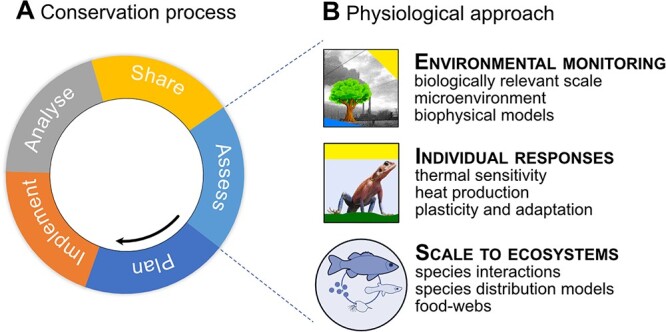
Summary of the interaction between conservation and physiology. The conservation process (A) as outlined in the *CS* comprises the sequential steps of assess, plan, implement, analyse, and share. Physiological research (B) can contribute to the assessment phase, and we suggest that the physiological approach comprises environmental monitoring, measuring individual responses to environmental change, and scaling these responses to ecosystem level to predict species distributions and changes to food web dynamics in response to climate warming, for example (all images by FS except for the clipart tree, which was used under a Creative Commons licence).

Conservation can have multiple goals, such as predicting threats and responses of conservation targets, removing threats and protecting vulnerable populations, geographical areas and ecosystems. Conservation typically follows a prescribed process: identification of challenges and goals, defining the spatial scale and actions, implementing actions and monitoring, and evaluation followed either by further updated rounds of the conservation process or by completion if goals have been achieved (Tallis *et al.,* 2021). This process of conservation is formalized in the CS designed by the Conservation Measures Partnership, which is composed of government agencies and nongovernment organizations from around the globe (https://www.conservationmeasures.org/). We focus on the CS here, which has been implemented in the context of climate change in the past (Brown *et al.,* 2022), but acknowledge that there are other conservation frameworks such as the Cambridge Conservation Forum (https://www.cambridgeconservationforum.org.uk/). The CS identifies and describes the steps that define the conservation process: assess, plan, implement, analyse and adapt, and share. The latest version of the CS includes several explicit climate change considerations (below we refer to these as *Climate Change Considerations*), to which the conservation physiology toolbox (Madliger *et al.,* 2018) can make important contributions (Tudor *et al.,* 2023). The assess step is the most important for incorporation of physiological responses, and the subsequent steps of planning and implementing will be guided by the physiological data. Below, we outline a conservation physiology approach that can contribute to positive conservation outcomes under climate warming. We divide the conservation physiology approach into three steps: environmental monitoring, individual responses and upscaling to ecological processes and ecosystems (Fig. 1). We point out how this approach integrates with CS *Climate Change Considerations* and provide brief examples where similar measures have already been implemented.

## Environmental monitoring

Identifying an appropriate geographical scale for conservation in the context of climate warming would almost always require assessment of the biophysical environment and the predicted shifts in the environment under different climate change scenarios. *Climate Change Consideration 1* emphasizes this need to define the scope of a conservation project and recognizes the difficulty that changing climates may alter the spatial extent of species ranges and ecosystems, thereby altering the geographical scope. The definition of geographical scope may therefore require repeated rounds of the conservation process (Fig. 1) (Tallis *et al.,* 2021). Geographical scope may be defined by different factors. For example, conservation of a defined area would set the geographical scope, and environmental monitoring would characterize that specific area. Conservation of particular species or ecosystems would define geographical scope by the presence or absence of those particular species or assemblages and would need to be repeated in changing climates.

The resolution at which environments are measured is crucial and must be biologically relevant (Helmuth *et al.,* 2014). Standard equipment for measuring temperatures, wind speed and solar radiation can be used to measure environmental variability and describe operative temperatures in local environments (Stupski and Schilder, 2021; Youngsteadt *et al.,* 2022) that influence individual and population level responses. For example, such environmental information can inform opportunities for behavioural thermoregulation in lizards (Buckley *et al.,* 2015). Although recorded at a local scale, these data can give valuable information about thermal habitat needs of individual species (Sears *et al.,* 2016; Basson *et al.,* 2017) that can be used in assessing the consequences of habitat modifications resulting from degradation or restoration. Using drones to map the physical environment of a rocky shore at fine resolution (2 ×2 cm) was the most effective scale to predict responses of intertidal organisms to climate change (Choi *et al.,* 2019). These microclimate data could then be integrated with physiological responses (e.g. respiration rate or heart rate) of resident organisms to thermal change to produce ‘physiological landscapes’ that permit modelling of species vulnerabilities to different scenarios of climate warming (Choi *et al.,* 2019). On the other hand, distributions or movement across large geographical scales, such as bird migration, requires modelling at a global level (Burnside *et al.,* 2021; Snell and Thorup, 2022)

Physiologically explicit modelling of different landscapes or geographical areas integrates environmental data with physiological responses to map fundamental niches of different species and at different scales (Kearney and Porter, 2016). ‘Niche Mapper’ is a tool developed for this purpose (Kearney and Porter, 2016) and is freely available (http://niche-mapper.com/). This biophysical niche modelling approach has been used very successfully to predict the efficacy of thermoregulation to buffer ectotherms from climate warming (Kearney *et al.,* 2009; Sunday *et al.,* 2014), model behavioural responses of a large mammal (moose, *Alces alces shirasi*) to climate variation (Verzuh *et al.,* 2023), assess heat stress in a vervet monkey (*Chlorocebus pygerythrus*) (Mathewson *et al.,* 2020) and assess the overwintering energetics of wood frogs (*Lithobates sylvaticus*) under climate warming (Fitzpatrick *et al.,* 2020), among many other applications. The strength of this biophysical niche modelling lies in the incorporation of specific physiological data, thereby linking environmental conditions explicitly to physiological responses (Briscoe *et al.,* 2023).

## Individual responses

*Climate Change Consideration 2* recommends an assessment of the extent to which climate change can impact the viability of conservation targets and of the efficacy with which conservation can improve performance of individuals and thereby population persistence of conservation targets. Environmental temperature changes impact physiological functions first and foremost. There is a plethora of laboratory studies that measured responses of many taxa to temperature variation (e.g. see database in Seebacher *et al.,* 2015). The most commonly measured physiological traits include rates of oxygen consumption as an indicator of energy use in ectotherms and of heat production potential in endotherms (Rummer *et al.,* 2014; Chouchani *et al.,* 2019; Norin and Metcalfe, 2019), mitochondrial bioenergetics to reflect cellular energy production (in the form of adenosine triphosphate) (Salin *et al.,* 2015; Treberg *et al.,* 2018; Sokolova, 2021) and aspects of muscle contractile function underpinning locomotor performance (James and Tallis, 2019). These physiological traits often scale up to influence energetics, growth and movement, which are central components in the ecology and therefore conservation of many species. Note, however, that not all individual traits have the same thermal sensitivities (Bozinovic *et al.,* 2020), and the choice of response measures is important. Whole-animal traits such as locomotor performance may be more suitable to assess thermal sensitivities than reductionist traits (e.g. single enzyme activities), because they integrate across physiological systems (e.g. cardiovascular system, metabolism and muscle function in the case of locomotion).

Mean temperature shifts and variability can cause chronic stress in wildlife that impacts performance and fitness (Skomal and Mandelman, 2012). These glucocorticoid-mediated stress responses support animals in coping with acute stressors through physiological and behavioural adjustments but may be detrimental in the long term (Schoenle *et al.,* 2021). Monitoring endocrine indicators of stress (e.g. glucocorticoid levels) is a useful and readily applicable tool to assess stress in wildlife that can be incorporated into conservation assessments (Narayan and Hero, 2014a, 2014b; Zimmer *et al.,* 2020; Schoen *et al.,* 2021; Schoenle *et al.,* 2021). However, the validity of using glucocorticoid concentrations as an indicator of stress, indicating decreased performance and fitness, should be assessed on a case-by-case basis because responses are not always consistent between and even within taxa (Jimeno *et al.,* 2018; Injaian *et al.,* 2020).

### Responses to warming

The impacts of increasing body temperatures range from modifying biochemical reaction kinetics to breaking down membranes and proteins, and different groups of organisms have quite different responses to temperature (Tattersall *et al.,* 2012). In ectotherms, environmental temperature can determine body temperature directly. In heterogeneous environments, thermoregulation by habitat selection and cardiovascular adjustments in ectotherms (e.g. in reptiles) and endotherms (e.g. birds and mammals) buffers the internal environment from external fluctuations (Angilletta, 2009), but only up to a point. Behavioural thermoregulation requires sufficient environmental heterogeneity to permit selection of favourable thermal habitats (Angilletta *et al.,* 2002). Endotherms can additionally thermoregulate by changing metabolic heat production (Chouchani *et al.,* 2019). Most biological reaction rates are sensitive to changes in temperature variation. Understanding the thermal sensitivity of physiological processes on one hand, and the potential for thermoregulation to maintain relatively stable body temperature on the other, is therefore essential to assess habitat quality for conservation. The range of temperatures at which animals perform well is defined by the thermal performance breadth in ectotherms (Sinclair *et al.,* 2016), and the thermal neutral zone in endotherms defines the range of temperatures at which metabolic heat production is minimized (Chouchani *et al.,* 2019). The temperature extremes that organisms can withstand before cellular integrity is compromised are defined by their thermal tolerance range, which is bounded by critical thermal limits in ectotherms (Gunderson and Stillman, 2015; Tomlinson, 2019). The thermal sensitivity of physiological rate functions is not fixed within organisms but can change with ontogeny or prior experience, for example (Sinclair *et al.,* 2016). Nonetheless, physiological thermal tolerance can be linked to patterns of endemism, and species or populations with narrow tolerance bounds can be constrained to small distributions that match these limits (Huey *et al.,* 2009; Rummer *et al.,* 2014). With climate warming, these species are expected to be most vulnerable to extinction as their suitable habitat and distributions contract to higher altitudes or latitudes, ultimately resulting in their being ‘pushed off the top of the mountain’ (Elsen and Tingley, 2015).

Climate warming may compromise thermoregulation by reducing the availability of suitable (cool) microhabitats for behavioural thermoregulation (Kearney *et al.,* 2009) and by increasing the need for evaporative cooling in endotherms (McKechnie *et al.,* 2016). Evaporative heat loss requires access to water, and as temperatures increase and available surface water decreases with climate warming, thermoregulation can become unattainable, ultimately leading to mortality of birds and mammals (McKechnie *et al.,* 2021). Effective biodiversity conservation for many birds and mammals therefore requires knowledge of the relationship between metabolic heat production and thermal tolerance on the one hand, and the efficacy of evaporative heat loss in the context of habitat features such as available surface water on the other (Mitchell *et al.,* 2018; Conradie *et al.,* 2020). This codependence of physiology and ecology is not restricted to conservation problems in hot arid areas. In the snow bunting (*Plectrophenax nivalis*), an Arctic songbird, metabolic and evaporative heat loss data indicate that global warming has already reached levels where the species must limit its activity levels to reduce metabolic heat production, which in turn is associated with reduced reproductive success (O'Connor *et al.,* 2022). Indeed, this is another example where effective conservation is contingent on detailed physiological knowledge to identify upper temperature thresholds and habitat requirements for different species and populations.

### Phenotypic plasticity and adaptation

Adaptation by natural selection is fundamental to how organisms evolve in response to environmental change. However phenotypic variation is more complex than just intergenerational change in response to selection pressures or genetic drift, and plasticity of physiological traits is a widespread response to environmental variability (Guderley, 2004; Schulte *et al.,* 2011). Plasticity may be induced by parental effects on their gametes (transgenerational plasticity), conditions experienced during early development (developmental plasticity), or in response to environmental changes at the scale of weeks or longer in adult organisms (reversible acclimation) (Shama *et al.,* 2014; Burggren, 2018; Loughland *et al.,* 2021). Plastic responses to temperature change are much quicker than genetic adaptation, and developmental plasticity, for example, can be mediated by epigenetic changes such as DNA methylation (Loughland *et al.,* 2021). Different forms of plasticity can thereby alter how well animals perform in different and changing environments and may buffer organisms from the impacts of climate warming to a certain extent (Gunderson and Stillman, 2015; Seebacher *et al.,* 2015; Fox *et al.,* 2019). It is therefore important to incorporate plastic responses and adaptation into predictive models such as species distribution models (see below).

## Upscaling to ecology and ecosystem function

*Climate Change Consideration 3* recommends the need for vulnerability assessments to determine the extent to which climate change can cause new threats or interact and exacerbate existing threats. Physiological knowledge of individual responses and upscaling these to ecosystem-level analyses and predictions can quantify how closely species operate to their optimal performance breadth currently and under future climates, and how higher-level interactions are likely to change (Seebacher and Franklin, 2012). Analysing climate predictions in the context of this physiological knowledge provides a data-driven assessment of the threats that climate change poses, particularly for ecosystems that are already under threat from overexploitation (Gaines *et al.,* 2018). Species distribution models are an essential tool for extinction risk analysis, and incorporating physiological data into models generally improves the accuracy of predictions of current and future suitable ranges of individual species or ecosystems (Evans *et al.,* 2015; Mathewson *et al.,* 2017; Tomlinson *et al.,* 2018). We have already described how physiological data can be incorporated into predictive models such as biophysical models [e.g. Niche Mapper (Kearney and Porter, 2016)]. These models can be used to predict species distributions based on their fundamental (physiological) niches. A future challenge will be to incorporate plastic responses into mechanistic species distribution models. Phenotypic plasticity and adaptation can broaden the range of suitable environments, and plasticity may buffer organisms from environmental variation up to a point (Seebacher *et al.,* 2015). The relatively rapid plastic responses to environmental variation and, in specific cases, of genetic adaptation (Lescak *et al.,* 2015) may render populations less vulnerable to climate warming (Seebacher *et al.,* 2015; Bush *et al.,* 2016). A recent species distribution modelling approach (ΔTraitSDM) incorporates adaptation and plasticity (Garzón *et al.,* 2019) and confirms that these evolutionary responses to environmental change can have beneficial effects on species distributions. It is therefore desirable to incorporate physiological plasticity and adaptation into species distribution models to improve the accuracy of conservation assessments.

### Trophic interactions and food web dynamics

In addition to altering suitable habitat availability, climate warming can also disrupt interactions between species via differential effects on their physiology (Van der Putten *et al.,* 2010). For example, different responses to warming changed the relative swimming performance of predator and prey species and thereby reduced the likelihood of prey being captured at higher temperatures (Grigaltchik *et al.,* 2012). Such temperature-induced mismatches in physiological rates between species can fundamentally change food web dynamics (Bideault *et al.,* 2020; van Moorsel *et al.,* 2023). Additionally, trophic transfer efficiency is projected to decrease with climate warming (Pontavice *et al.,* 2021). For example, in zebrafish, the food-derived energy used to produce a given amount of new biomass (energetic cost of growth) rose sharply with an increase in temperature from 25°C to 32°C (Barneche *et al.,* 2019). Using nitrogen transfer as an indicator of energy transfer, an increase of 4°C in water temperature reduced growth efficiency by 56% in a long-term mesocosm experiment with plankton communities (Barneche *et al.,* 2021). These temperature effects on interacting species within food webs are driven by the thermal sensitivity of underlying physiological rates (Sokolova, 2021; van Moorsel *et al.,* 2023; Wootton *et al.,* 2023), and physiological data (e.g. metabolic rates and growth rates) can complement ecological analyses to lead to more accurate assessments of changes in food web dynamics and trophic cascades (Galiana *et al.,* 2021).

Ecosystem level responses to climate warming and associated extreme events can have pronounced impacts on human societies. Disruption of food web structures and trophic interactions affect the relative abundance of different species within ecosystems with potentially negative impacts on food security (Beas-Luna *et al.,* 2020). Changes in species distribution can alter availability of food species directly (Yang *et al.,* 2022), or they can alter the availability of ecological services such as pollination (Pyke *et al.,* 2016; Tomlinson *et al.,* 2018), both of which can affect food security. Additionally, the physiological effects of warming on individuals can negatively impact the sustainability of wild harvests. For example, recreational fishing with rod and reel is a popular activity around the globe, and even though it is not ‘essential’ for food supply, it nonetheless has major impacts on target species. Although a portion of fish caught by recreational anglers are harvested, even more (~70%) are released, equating to billions of fish each year (Cooke and Cowx, 2004). The premise of catch-and-release fishing is that most fish survive, although that is not always the case. Water temperature is a key factor influencing the fate of fish that are caught and released (Gale *et al.,* 2013). When fish are caught at ‘high’ (relative for a given population) temperatures, physiological stress responses and exhaustion are likely and may lead to unintended mortality (Holder *et al.,* 2022). Recreational fishing mortality has increased with climate warming, which has elicited a range of management responses that restrict fishing (Jeanson *et al.,* 2021). Already there are water temperature thresholds that if exceeded lead to the closure of some high-value fisheries as a result of physiological dysfunction (Wilkie *et al.,* 1997; Lennox *et al.,* 2017; Van Leeuwen *et al.,* 2020). Knowledge of these physiological sensitivities has guided conservation interventions, and different jurisdictions have enacted various triggers to close rivers for fishing that reflect population-level thermal thresholds (Van Leeuwen *et al.,* 2020).

A synthesis between physiology, distribution models and climate predictions can feed into the conservation planning process to attain conservation goals in the context of current and future climate warming (*Climate Change Consideration 4*). Ultimately, assessment and planning must lead to conservation interventions to achieve the conservation goal. Detailed physiological knowledge of sensitivities to temperature change will benefit climate-related conservation strategies provided that such information is shared with conservation managers in relevant formats (Laubenstein and Rummer, 2021). Identifying climate refugia, creating artificial habitat, or enhancing the viability of a conservation target are suggested in *Climate Change Consideration 5* as potentially effective conservation strategies. Knowledge of physiological sensitivities to temperature change can be invaluable to test the efficacy of these interventions. For example, the effects of habitat restoration or creation of new habitat features to provide suitable thermal habitats can be assessed directly from laboratory studies testing thermal responses of target species. Climate warming may alter environments in protected areas so that their habitat characteristics no longer match the requirements of conservation targets (Araújo *et al.,* 2011; Basen *et al.,* 2022). While protected areas remain valuable and necessary (LeDee *et al.,* 2021; Rummer *et al.,* 2022), they may not always be sufficient (Fernando and Pastorini, 2021; LeDee *et al.,* 2021; Moore and Schindler, 2022). Landscapes worked by humans (e.g. urban and agricultural landscapes) can also provide important habitats for wildlife and harbour functioning ecological communities (Fahrig *et al.,* 2011; Pedroza-Arceo *et al.,* 2022). Physiological assessments can offer an effective approach to identify the conservation value of different environments by mapping environmental conditions (e.g. heterogeneity of thermal habitats) to physiological performance (e.g. thermal sensitivity of locomotion and other performance measures). The utility of physiological data thereby extends beyond individual species to habitat conservation and biodiversity. More complex habitats also support a broader range of species and thereby improve biodiversity (Wild *et al.,* 2011; Sato *et al.,* 2014; Hekkala *et al.,* 2023). Complexity and heterogeneity of habitats are therefore essential criteria to establishing novel ecosystems, for example, ecosystems created in urban environments, which can be an effective tool for maintaining biodiversity (Ignatieva *et al.,* 2023). Knowledge of physiological sensitivities (e.g. thermal sensitivity) of key biodiversity components is important to inform establishment of appropriate habitat features (Sato *et al.,* 2014).

## Summary and conclusions

This *Perspective* has focused particularly on the impacts of climate warming. However, the impacts of climate change are much broader and encompass changes in rainfall and drought, ocean acidification and impacts on nutritional environments, for example (Pörtner *et al.,* 2022). A more comprehensive review was beyond our scope, but a similar approach to the one we describe here to assess the impacts of warming could also be applied to changes in other environmental variables. Enlisting physiology, ecology (including demography and behaviour) and genetics together will inform the development of the most robust conservation decisions and interventions. Physiology can detect the sensitivity of individuals to environmental change and assess the potential for populations to respond to change via phenotypic plasticity (Seebacher and Franklin, 2012; Fox *et al.,* 2019); genetic research can determine mutation rates and changes in allele frequencies to assess the potential for genetic adaptation in responses to environmental change (Lescak *et al.,* 2015; McGaughran *et al.,* 2021); physiological and genetic insights can contribute to ecological analyses of higher-level responses and interactions (Loria *et al.,* 2022; Wootton *et al.,* 2023), and estimates of rates of ecological change in the face of climate change (Williams *et al.,* 2021). Such integrated mechanistic approaches to conservation are lacking (Cooke *et al.,* 2023) despite great potential to ensure that conservation actions are targeted and effective.

How can physiology be integrated into the conservation process? Conservation is a political process, to a large extent (Büscher and Fletcher, 2019), and funding may be allocated for reasons other than solely ecological value. Nonetheless, the responsibility for biology and its practitioners lies in providing the best possible assessment of conservation problems to lead to the most effective conservation outcomes given financial and other constraints. To achieve this, biological assessments need to be inclusive. Physiology is part of this assessment. Much of the needed physiological knowledge is already in the literature so that evidence syntheses (Cook *et al.,* 2017) are a first step in incorporating physiological knowledge into conservation, particularly by higher-level organizations such as government institutions and global NGOs that have access to a broad range of evidence and the expertise to interpret and synthesize it. Bespoke physiological knowledge to address specific conservation problems can be generated by research funding by government and government–industry or government–NGO partnerships. Physiological data generation may be perceived to be complicated and restricted to specialist laboratories. However, there are several widely accepted physiological measurements (Madliger *et al.,* 2018) that are relatively easy to collect in the field at a local scale to determine thermal sensitivities of particular populations, for example. Together with ecological and genetic techniques, these approaches can provide effective conservation assessment that will enable evidence-based conservation and environmental management.

Areas for future research include broader geographical coverage. Most research on physiological responses to environmental variation has focused on Europe and North America, and there are next to no data for geographical areas of high biodiversity in Africa and South America, for example (White *et al.,* 2021). Similarly, there are taxonomic biases (Palma *et al.,* 2016; Dornburg *et al.,* 2017) that limit the generality of current understanding how wildlife responds to environmental change. Finally, treatment conditions in experimental studies often do not represent natural conditions so that experimental insights, while being conceptually important, may have limited utility for conservation (Morash *et al.,* 2018; Hall and Warner, 2020).

## Funding

This work was supported by the Australian Research Council (DP220101342 to F.S.), the Australian Research Council Centre of Excellence for Coral Reef Studies (to J.L.R.), the Natural Sciences and Engineering Research Council of Canada (D.G. to S.J.C) and Genome Canada via the GenFish project (to S.J.C).

## Data availability

There are no data associated with this article.
